# Ankle Robotics Induces Ongoing Locomotor Plasticity with Delayed, Sustained Multi-Segmental Gait Improvements 17 Months After Training in Chronic Stroke

**DOI:** 10.3390/medicina62071250

**Published:** 2026-06-29

**Authors:** Anindo Roy, Kelly Westlake, Charlene Hafer-Macko, Bradley Hennessie, Richard Macko

**Affiliations:** 1 NextStep Robotics, Inc., MDC Studios, 300 West Pratt Street, Ste 200, Baltimore, MD 21201, USA; brad.hennessie@nextsteprobo.com; 2 Maryland Robotics Center and Maryland Applied Graduate Engineering (MAGE), University of Maryland, College Park, MD 20742, USA; 3 Department of Physical Therapy and Rehabilitation Science, University of Maryland, Baltimore, MD 21201, USA; kwestlake@som.umaryland.edu; 4 University of Maryland Older Americans Independence Center, Department of Medicine, Geriatrics, University of Maryland School of Medicine, Baltimore, MD 21201, USA; 5 Fleni Neurological Institute, Buenos Aires C1428AQK, Argentina; chmacko@gmail.com; 6 Solutions for Developing Countries, University of West Indies, Mona, Kingston 7, Jamaica

**Keywords:** stroke rehabilitation, ankle robotics, hemiparetic gait, delayed locomotor plasticity, gait biomechanics, fall-risk/gait safety, long-term outcomes, adaptive control robotics, robotics-assisted gait training, emergent neuroplasticity

## Abstract

*Background and Objectives*: Robotics training improves gait after stroke, but no prior studies have investigated whether emerging long-term gait biomechanics improvements occur after training. We assessed the temporal profile of pre-post gait biomechanics changes after 9 weeks of dorsiflexion specific adaptive control ankle robot (AMBLE™) training, and at 9 weeks post-training and 17 months later in three persons with chronic stroke to probe for ongoing locomotor plasticity versus post-training disuse decay. *Materials and Methods*: Three densely hemiparetic subjects (mean ± SD), age 62 ± 7 years., stroke latency 8 ± 4 years, available for repeat testing from an original N = 24 robotics training cohort study, underwent three-dimensional gait analyses pre-post 9 weeks of AMBLE training, and then 9 weeks and 17 months after all robotics training ended. *Results*: We found that only 47% of total improvements in heel-first strikes and 31% increased paretic step length occurred pre-post training. Unexpectedly, all other biomechanical improvements manifested progressively 17 months after training ended, including ankle peak swing angle (∆ = 7°), dorsiflexion angular velocity (∆ = 23°/s), peak knee flexion (∆ = 11.1°) and hip flexion (∆ = 6°). Robotics prescription progressions in level of assistance and dorsiflexion target angle strongly correlated to gait biomechanical outcomes at 17 months, including improved heel-first strikes and peak dorsiflexion swing angle in this small sample. *Conclusions*: These findings show that initial improvements in foot–ankle function across training are followed by emergent biomechanical improvements in ankle, knee and hip kinematics across 17 months post-training, with delayed outcomes related to robotics prescription progression. The temporal profile of biomechanical adaptations might suggest delayed, progressive reduction in pathological multi-joint synergies of the hemiparetic leg. However, findings are exploratory and cannot establish causality, treatment efficacy or broad generalizability. Future research is needed to determine whether ankle robotics training can catalyze improvements in long-term gait biomechanical safety and efficiency in the chronic disease management of stroke.

## 1. Introduction

Stroke is a leading cause of physical disability. Hemiparetic gait is the most prevalent neuromotor deficit pattern to persistently impair the safety and efficiency of walking and balance functions [1,2,3,4]. Foot drop affects approximately 30 percent of persons with more severe hemiparesis and heightens fall risk [5]. Inadequate toe clearance typically results from abnormal multi-segmental motor control of ankle, knee and hip motor kinematics [6,7,8]. Robotics-assisted gait training has emerged as a promising avenue to improve elements of gait biomechanics for persons with hemiparesis including those with foot drop [9,10,11,12]. Ankle robotics gait training studies in persons with hemiparesis have primarily reported the immediate pre-post training changes and focused on foot–ankle biomechanics and standardized function mobility outcomes, with only limited studies showing longitudinal durability of functional gains [9,10,11]. No prior studies, to our knowledge, have investigated emerging multi-segmental biomechanical improvements this long after robotics training has ended in persons with chronic hemiparesis after stroke. Chronic stroke is the preferred population for testing ongoing post-training locomotor plasticity. The chronic phase of stroke, especially in cases nearly a decade after the index disabling event as in these participant cases, is generally considered to be neurologically plateaued in the absence of further interventions [5]. Furthermore, functional ambulatory capacity is expected to decline across years secondary to aging with the chronic disability of stroke [13,14]. Thus, there is a knowledge gap regarding whether ankle robotics-assisted gait training can durably, or even continuously improve gait biomechanics in chronic hemiparetic stroke. The latter of which would suggest ongoing locomotor plasticity unexpectedly in the chronic stroke population.

We recently reported that gait training with a modular robotic ankle (AMBLE™ NextStep Robotics, Inc., Baltimore, MD, USA) durably improves ankle–foot, knee and hip kinematics, as well as clinical measures of functional mobility in N = 24 persons with chronic hemiparetic stroke and foot drop [15]. Notably, 9 weeks of two times per week (30 min per session for 18 sessions) AMBLE training produced pre-post training improvements in functional mobility outcomes but only selected biomechanical improvements in gait, exclusively at the ankle–foot kinematic level. Gains included toe clearance, ankle dorsi- and plantar-flexion angular velocity, paretic step length and heel-first strikes landings. The improvements higher up the kinematic chain in hip and knee flexion manifested only at the durability testing timepoint, 9 weeks after robotics training ended [15]. These findings suggested that adaptive control ankle-robotics-assisted gait training produces a temporal profile of biomechanical adaptations with initial end-effector improvements primarily across the ankle, followed by delayed improvements in multi-segmental gait biomechanics up the kinematic chain. Building on our prior study, this brief report investigated the long-term (17 months after training ends) delayed biomechanical adaptations in a convenience subset of three individuals with chronic hemiparetic gait and foot drop. These participants that completed AMBLE training returned for a different research study after an extended follow-up without interim rehabilitation or clinical trial participation. We hypothesized based on the natural history of aging with the chronic disability of stroke that there would be incremental deterioration of robotics training effects and/or adoption of compensatory strategies, leading to an overall clinical worsening and reduced safety of gait biomechanics over the 17 months without further formal gait training.

## 2. Materials and Methods

Design: This analysis was an exploratory follow-up to our previously published AMBLE trial [15]. In that parent, single-arm study, 24 participants with chronic hemiparetic gait and foot drop after stroke completed 9 weeks of AMBLE training with baseline, post-training and 9-week retention outcome assessments. This original research was funded by the National Institute of Health (NIH): National Institute of Neurological Disorders and Stroke (NINDS) under cooperative research agreement 5U44NS111076 “Portable Ankle Robotics to Reverse Foot Drop After Stroke.” NIH funding approval date was 20 August 2020. This study (NCT04594837) was registered on the ClinicalTrials.gov site on 14 October 2020. The NIH U44 research grant had two phases. The U44 phase one focused on engineering development. Normally, this was a one-year process; we had an additional year for a no-cost extension due to the delays that resulted from shut-downs affecting the supply chain during the COVID-19 global pandemic. U44 phase two was clinical testing; phase two funding started two years later in 2022. The ethical approval date issued by the Salus Institutional Review Board was 18 October 2022. All participants in the parent NIH U44 trial provided written informed consent, and the protocol was approved by Salus Institutional Review Board (Salus 22226-1U44NS111076-01; Portable Ankle Robotics, approved 18 October 2022). As per Research Code of Ethics and Regulations and Good Clinical Practice, the clinical trial was registered prospectively, and research ethics approval was obtained before the enrollment of the first participant to ensure transparency and scientific integrity.

The 17-month testing for these three participants was obtained from baseline testing for a second study. University of Maryland Baltimore Human Research Protection Office approved the protocol and the informed consent form (HP-00108406) on 31 May 2024. Data from this second study served as pilot data for a NIH RO1 submission. All research participants provided written informed consent approval and met inclusion and exclusion criteria in the two approved research protocols.

Baseline evaluations included the review of medical records, imaging, medical and neurological exams and cardiopulmonary clearance to establish eligibility and characterize neurological deficits. The original NIH U44 study had no control group. To establish efficacy in this chronic stroke population, subjects served as their own pseudo-controls with double baseline outcome testing conducted at least one week apart. Eligible participants received 9 weeks of AMBLE therapy (two 30 min sessions per week of combined treadmill and overground walking). Outcome testing was conducted after 9 weeks of ankle robot walking training, and again 9 weeks after all training ended. A letter explaining the new research study was mailed to all subjects from the original NIH U44 cohort. They were provided with the new study team contact information. They were advised to contact the team should they be interested in participating. This new unfunded research study sought preliminary data from only a limited number of subjects in preparation for a NIH RO1 grant re-submission.

For the present brief report, we included a convenience subset of three participants from the parent cohort who: (i) had completed all parent-study assessments, and (ii) subsequently consented to participate in a separate second rehabilitation study (HP-00108406). The three participants provided additional written informed consent for the University of Maryland Baltimore Institutional Review Board protocol (HP-00108406) that was approved on 31 May 2024. Baseline gait biomechanic outcomes for this second study provided our additional 17-month durability assessment. No participation in clinical research trials or any additional formal rehabilitation was delivered between these 9-week and 17-month post-robot training assessments.

Apparatus: The AMBLE device was previously described in detail [15]. Briefly, it was a lightweight (1.4 kg), wearable, portable, Bluetooth operatable, ankle exoskeleton worn unilaterally on the paretic limb. Adaptive impedance control was delivered precisely timed for dorsiflexion assistance across three swing phase sub-events. The three dorsiflexion sub-events were the rise phase (post toe-off to peak swing), mid-swing hold and foot descent (controlled dorsiflexion at initial contact to mitigate foot slap) [16,17]. Its untethered design permitted overground and treadmill walk training. The device had a single degree-of-freedom (DOF) actuation but permitted normal ankle range of motion in all three DOFs. It generated up to 20 Nm of torque at the ankle, which was sufficient for adequate ground clearance during limb swing.

Inclusion/Exclusion Criteria: Inclusion criteria were the same for both the parent [15] and second protocol. Criteria consisted of: (i) unilateral ischemic or hemorrhagic stroke; (ii) >6 months post-stroke; (iii) age > 18 years; (iv) medical clearance; (v) residual hemiparesis of the lower extremity, as previously defined; (vi) completed conventional therapy; and (vii) the ability to ambulate at least five feet without an ankle foot orthosis (AFO) with their usual cane or walker, but with no more than minimal contact assistance. Exclusion criteria consisted of: (i) unstable angina, recent myocardial infarction, congestive heart failure or hemodynamically significant valvular dysfunction; (ii) uncontrolled hypertension (>160/100 on two assessments); (iii) recent hospitalization (<3 months); (iv) symptomatic peripheral arterial occlusive disease; (v) orthopedic, chronic pain or non-stroke neuromuscular disorder restricting gait; (vi) active cancer; and (vii) aphasia or cognitive functioning that confounded adequate informed consent and safe participation (e.g., unable to communicate discomfort or unable to follow 2-step commands).

Outcome Testing: Research laboratory outcome testing included a timed 10 m walk (gait velocity); manual motor testing of ankle dorsiflexion; ankle active and passive range of motion; and gait biomechanics acquired using a motion capture system (Vicon™, Oxford, UK) during unassisted overground walking. Primary outcome measures were peak dorsiflexion angle (degrees), dorsiflexion angular velocity and percentage of steps with heel first foot landings. Baseline testing was conducted twice, at least one week apart before all AMBLE gait training commenced. Post-testing was conducted after all the AMBLE training sessions were completed. Durability or retention testing was conducted 9 weeks after all training had ended. The three subjects in this brief report had subsequent durability tests at a mean of 17 months after training ended in the parent study [15]. This was the baseline timepoint for a separate rehabilitation study (HP-00084830). Thus, biomechanical and functional outcomes were assessed twice at baseline, post-training and then at the two retention timepoints (9 weeks and 17 months after training ends) to assess whether the training gains were durable.

Training Intervention [15]: Ankle-robot-assisted gait training was conducted at NextStep Robotics, Baltimore, MD, USA. Participants received a total of 18 supervised treadmill and overground walking sessions (goal of between 30 and 40 min actively walking with AMBLE assisted walking each session) across a 9-week time-period. AMBLE ankle robot training targeted specific sub-events of dorsiflexion (toe off to peak swing, hold and foot descent). Each session was scheduled for one hour. Pre-training activities included removal of any ankle foot orthosis device and robot donning and calibration. At the start of each daily robotic training session, subjects walked for 10 paretic steps with the robot in “evaluation,” “record-only” mode (no active assist). Ankle trajectories from these record-only trials were used to approximate the spatial (minimum angle following toe-off and peak swing dorsiflexion) and temporal (rise and hold times) parameters to characterize a nominal reference trajectory in the AMBLE software app to personalize daily training for each participant. These training input parameters were modified and progressed by the therapist/trainer, as needed, on-the-fly, across each session while walking on the treadmill and over-ground with the AMBLE device in active assist mode. Post-training activities included robot doffing, visual inspection of the paretic leg for redness or skin irritation and query for any adverse events, such as fatigue, chest pain, shortness of breath, dizziness, pinch points, device slippage, musculoskeletal or joint discomfort or discomfort due to the weight of the robot.

The design of the original robot-assisted training study was to improve ankle motor control in the context of a walking task. Both treadmill and over-ground walking had scripted recommendations for each subject to walk at their comfortable self-selected pace. Subjects were allowed to use their usual assistive devices (cane or walker) for overground safety. Assistive device usage during each training session was recorded. Handrail support was encouraged for safety while walking on the treadmill (Biodex GT3, Shirley, NY, USA). Exercise intensity was intentionally performed at a light exertion level (from 8 to 10) on the Borg Rate of Perceived Exertion (RPE, scale from 6 to 20). Exercise intensity was also tracked with a Polar heart rate monitor, Kempele, Finland) to assure that participants trained at a low aerobic exercise intensity, as defined according to the criterion of the American College of Sports Medicine. Each subject’s heart rate during training was held below 50% of their heart rate reserve, based upon the formula of Karvonen, and adjusted for any chronotropic medication usage (e.g., beta blockers), as by convention. Subjects were provided with interval rests, as needed. The time spent walking and resting was recorded as total therapy and total session times. Walking speed and exercise intensity were not advanced across the 18 walking training sessions.

Statistical Analysis: Because this was an exploratory add-on analysis in a convenience sample of three participants, analyses were descriptive statistics. We reported means ± SD at each time point and absolute changes between baseline, post-training, 9-week and 17-month retention visits. Given this small sample size, we did not use inferential statistics to make predictions or generalizations, this limits interpretation of the findings to the broader stroke population. Simple linear regressions were used to describe associations between changes in robot training input settings and gait outcomes, and coefficients of determination (r^2^) are reported as descriptive indices without formal hypothesis testing.

No generative artificial intelligence (GenAI) was used in this paper.

## 3. Results

### 3.1. Baseline Characteristics

**Figure 1:**
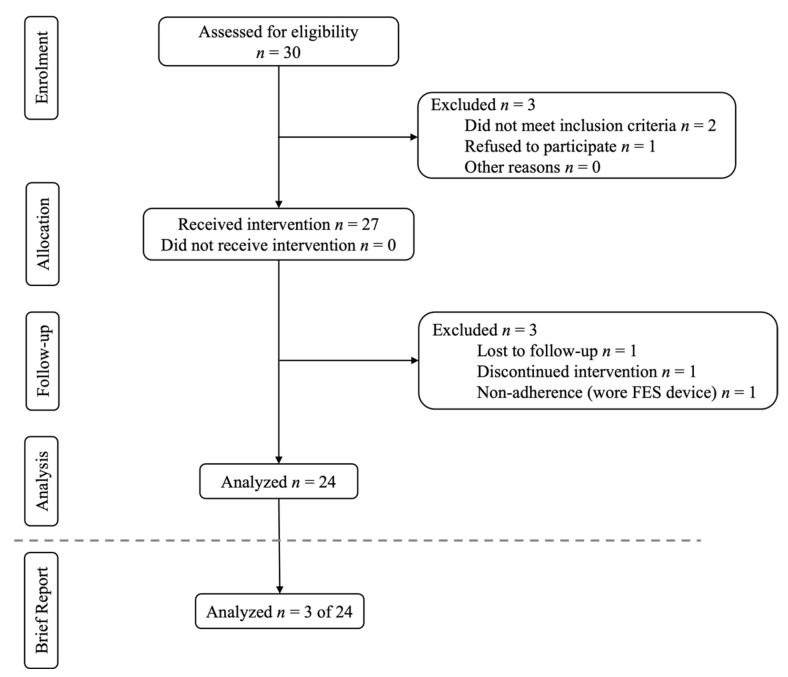
Consort flow chart [15].

Three participants (1 male, 2 females) from the original study cohort (N = 24) had additional testing 17 months after completing all robotics training (Figure 1: Consort Flow Diagram and Supplementary Table S1: Baseline Characteristics of the Original Cohort). Subject characteristics were as presented in Table 1. The mean ± SD subject age was 62 ± 7 years, and the time since stroke was 99 ± 52 months. Hemiparesis was on the left in all three. Manual muscle test dorsiflexion scores ranged from 1+ to 2+. Mean active and passive ankle dorsiflexion ranges of motion were −7 ± 6° and −8 ± 2°, respectively. Two subjects utilized an ankle–foot orthosis, and all three used a cane. At baseline testing, these three had Dynamic Gait Index (DGI) scores that were an average of 13.7 ± 1.5. All subjects had DGI scores < 16.5; thus, they were classified in a high fall risk category [18]. All baseline biomechanical outcome values for these three individuals were slightly lower compared to the average values for the full original cohort. The three participants had gait velocity that was slightly lower than the original cohort, but all three had moderate gait speeds that would be characteristic of limited community ambulators [19,20,21]. They had shorter paretic step length, and their peak dorsiflexion swing angle was just below neutral. Their peak dorsiflexion angular velocity was significantly less, and they had negligible heel first foot landings at baseline. The three had lower peak knee flexion angles but greater hip flexion at baseline compared to the full cohort.

### 3.2. Training and Nine-Week Retention Results

**Figure 2:**
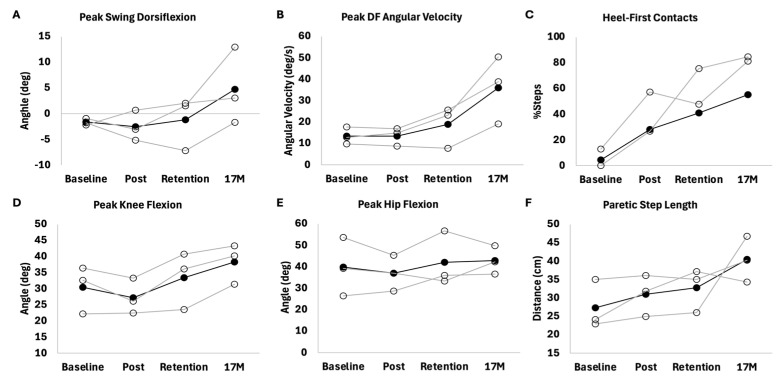
Changes in gait biomechanics in N = 3 subjects (each individual’s values across the four time points are indicated with open circles and a grey line; the average value for the three are indicated by closed circles and the dark black line) for baseline (average of two baseline tests), post-AMBLE training and two retention timepoints at 9 weeks and 17 months. Graphs illustrate the changes in (**A**) ankle dorsiflexion peak swing angle (degrees), (**B**) ankle peak dorsiflexion angular velocity (degree/seconds), (**C**) percentage of steps with heel first contacts (the third participant had none at any timepoint, so the value points are not shown), (**D**) peak knee flexion angle (degree), (**E**) peak hip flexion angle (degree), and (**F**) paretic step length (cm) across time.

AMBLE training improved the percentage of heel-first foot strikes in two of these three participants (Figure 2 and Table 2). The average pre-post gain in percent heel-first foot landings was +24 ± 16% steps. The training gain in heel-first foot strikes was only 12% in the original larger cohort, but the original cohort were less impaired with an average 32% heel-first stroke at baseline. The three participants also marginally improved their paretic step length (+4 ± 3 cm). This gain in paretic step length was of similar magnitude to the larger cohort (+3 cm). The three had no change in their self-selected overground gait speed. The larger cohort had a gain of 0.04 m/s after training. This gain in the larger cohort did not reach the meaningful difference level of between 0.05 and 1.0 m/s in comfortable gait speed, a change that exceeds what would be expected from normal day-to-day fluctuations and would signal early therapeutic progress, especially for slower walkers [20,21]. The three did not have a change in peak dorsiflexion angular velocity. The larger cohort had a significant average training gain in dorsiflexion angular velocity (+12°/s), but they were much less impaired at baseline. The three had a negligible gain observed for peak ankle dorsiflexion swing angle, and slight worsening in peak knee and hip flexion angles.

The nine-week retention testing revealed continued gains after all training ceased in the three participants. At the nine-week retention timepoint, the percentage of heel-first foot strikes increased to 41 ± 38%; this represented a continued gain of +13 ± 24% steps with safer landings. Paretic step length increased modestly (+2 ± 2 cm) during the retention period. Compared to baseline values, paretic step length increased by 5.5 cm ± 5.2 cm; this approaches the reported mean clinically important difference (MCID) of 6.8 cm [22,23]. The larger cohort had a similar gain in paretic step length after training and during the retention period. The three participants had a modest gain in their self-selected overground gait speed (+0.04 ± 0.05 m/s) during retention. The larger cohort had a similar retention period gain in velocity of +0.03 m/s. The peak dorsiflexion ankle angle increased by (+1.2 ± 2.2°) during the retention period. Peak dorsiflexion angular velocity modestly improved (+5 ± 6°/s). Moderate increases in untargeted knee (+6 ± 5°) and hip (+5 ± 8°) flexion were observed during retention in the three participants, and their gains were slightly greater than the average gains in the larger cohort (peak knee flexion was + 4° and hip flexion +2°).

**Table 2:** Gait biomechanics outcomes at the dual baseline, post-training and the 9-week and 17-month retention time points for the three subjects and the larger cohort (N = 24).

Outcome Measure and Time	CPTR16	CPTR20	CPTR25	Mean ± SD N = 3	∆	Mean ± SD Original N = 24	∆
Peak swing DF (°, normal +10°) [24]							
Baseline 1	−1.6	−0.2	−1.9	−1.2 ± 0.4		0.5 ± 6.5	
Baseline 2	−1.9	−1.7	−2.6	−2.1 ± 0.4		0.2 ± 6.4	
Post-train	−5.2	−3.1	0.7	−2.5 ± 1.7	+0.9	0.5 ± 6.4	+0.2
Retention 9-week	−7.2	1.5	2.0	−1.2 ± 3.0	−1.2	1.6 ± 7.1	+1.4
Retention 17-month	−1.7	12.9	3.0	4.7 ± 4.3	+7.2		
Peak DF angular vel (deg/s, healthy 100–150°/s, chronic stroke 42°/s)							
Baseline 1	10	14	15	13 ± 2		34 ± 29	
Baseline 2	9	12	20	14 ± 4		37 ± 37	
Post-train	9	15	17	14 ± 4	0	47 ± 40	+12
Retention 9-week	8	23	26	19 ± 10	+5	43 ± 38	+7
Retention 17-month	19	50	39	36 ± 16	+22		
Heel-first strikes (% steps; goal 100%)							
Baseline 1	0	0	15	5 ± 7		31 ± 41	
Baseline 2	0	0	10	3 ± 4		32 ± 42	
Post-train	0	27	57	28 ± 29	+24	44 ± 43 ^†^	+12
Retention 9-week	0	75	48	41 ± 38	+13	46 ± 43	+14
Retention 17-month	0	85	81	55 ± 48	+27		
Peak knee flexion (°, MCID +4°) [25]							
Baseline 1	22.5	30.9	33.6	29.0 ± 7.4		32.2 ± 16.9	
Baseline 2	21.9	34.2	39.2	31.8 ± 6.6		34.0 ± 16.6	
Post-train	22.4	26.1	33.2	27.3 ± 5.5	−3.1	35.0 ± 16.2	+1.9
Retention 9-week	23.5	36.1	40.8	33.5 ± 8.9	+6.2	36.9 ± 17.7 *^,‡^	+3.8
Retention 17-month	31.4	40.2	43.4	38.3 ± 6.2	+11.0		
Peak hip flexion (°, MCID +5.8°) [26]							
Baseline 1	26.5	53.7	39.2	39.8 ± 9.3		36.9 ± 9.7	
Baseline 2	24.5	53.4	39.1	39.0 ± 9.7		35.9 ± 9.3	
Post-train	28.7	45.4	36.9	37.0 ± 8.3	−2.4	37.8 ± 8.2	+2.4
Retention 9-week	36.0	56.7	33.3	42.0 ± 12.8	+5.0	39.6 ± 9.2 *	+1.8
Retention 17-month	36.6	49.7	42.2	42.8 ± 6.6	+5.8		
P-step length (cm, MCID 6.8–8.1 cm) [22,23]							
Baseline 1	34	25	26	28 ± 4		37 ± 16	
Baseline 2	36	23	20	26 ± 6		37 ± 16	
Post-train	36	32	25	31 ± 6	+4	40 ± 14 ^†^	+3
Retention 9-week	35	37	26	33 ± 7	+2	39 ± 13	+2
Retention 17-month	40	34	47	40 ± 6	+9		
Gait velocity (m/s, MCID 0.05–0.10 m/s) [20,21]							
Baseline 1	0.6	0.51	0.57	0.56 ± 0.03		0.64 ± 0.24	
Baseline 2	0.52	0.47	0.57	0.52 ± 0.03		0.67 ± 0.25	
Post-train	0.7	0.47	0.51	0.54 ± 0.09	0	0.70 ± 0.23 ^†^	+0.04
Retention 9-week	0.63	0.59	0.53	0.58 ± 0.05	+0.04	0.69 ± 0.23	+0.03
Retention 17-month	0.5	0.46	0.57	0.51 ± 0.06	−0.03		

∆ are (a) avg baseline-post and (b) post-9-week durability for the three individuals and the original cohort; and (c) post-17-month durability for the three individuals. MCID = meaningful clinical important difference. MDC = minimal detectable change. * *p* < 0.05, ^†^ *p* < 0.01, ^‡^ change > MCID.

### 3.3. Delayed Adaptations at the 17-Month Retention Timepoint

The three participants demonstrated continued improvements in selected measures at 17 months’ retention, beyond the gains at the 9-week retention period (Figure 2, Table 2). At 17 months, further gains, beyond the 9-week retention timepoint, were observed in peak swing dorsiflexion (+6 ± 5°), dorsiflexion angular velocity (+17 ± 9°/s) and heel-first foot strikes (+14 ± 17% steps). Delayed proximal joint kinematic improvements included gains in peak knee flexion (+5 ± 3°), though hip flexion remained unchanged. Paretic step length increased (+9 ± 6 cm). Interestingly, walking speed decreased slightly (−0.03 ± 0.05 m/s). Importantly, compared to average baseline values for these three individuals, there were substantial improvements observed in peak swing dorsiflexion (+6 ± 7°), dorsiflexion angular velocity (+23 ± 14°/s), heel-first foot strikes (+51 ± 45% steps) and paretic step length (+15 ± 10 cm). The paretic step length gains from baseline exceeded the reported MCID (from 6.8 to 8.1 cm) [19,20]. These changes were accompanied by increased knee (+8 ± 1°) and hip (+3 ± 7°) flexion. The increase in peak knee flexion was greater than the reported mean clinically important difference (MCID) (+4°) [25], and hip flexion approached the MCID (+5.81°) [26]. Surprisingly, with these biomechanical gains, walking speed declined.

### 3.4. Relative Gains in Gait Biomechanics Across Robotics Training Versus After Training Ends

**Figure 3:**
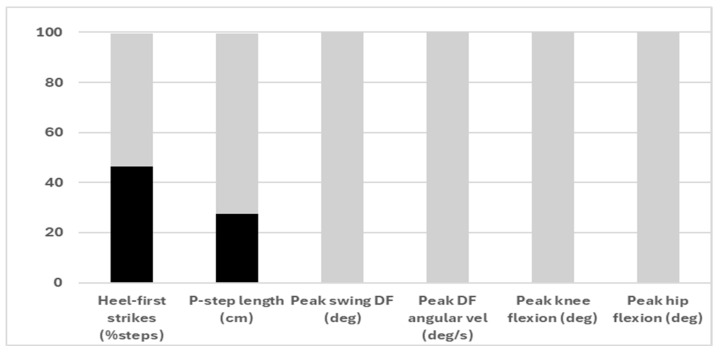
The magnitude and percentage of gain in biomechanical outcome from the training versus delayed gains after completion of all training during the 17-month total retention period (P: paretic). The black bars signify the percentage of changes that occur across the 9 week ankle robotic training, while the grey bars signify the percentage of changes across the 17 months after all training ends.

Comparison of the absolute and relative percent changes that occurred immediately from baseline-to-post training versus across the 17-month total retention period after training ended showed that the majority of gait biomechanical improvements manifest long after robotics training has ended. Specifically, all of the improvements in unassisted overground paretic peak swing dorsiflexion, peak knee flexion and hip flexion occurred at the 17-month retention timepoint. In relative terms, nearly half of the total improvement in heel-first strikes and nearly one-third of the total improvement in paretic step length occurred immediately after training (Figure 3). The only variable that showed a decline at the 17-month retention timepoint was gait velocity; it declined back toward the baseline value. The immediate robotics training mediated improvements were categorically in selected paretic foot landing and gait temporal-distance parameters, while kinematic improvements in the paretic ankle, knee and hip manifested 17 months after training ended in this small cohort.

### 3.5. Temporal Profiles of Training Inputs

**Figure 4:**
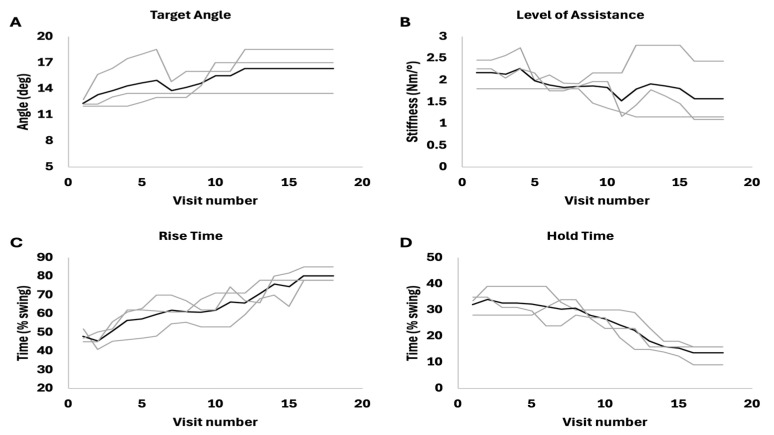
AMBLE robotic training parameter inputs for each individual (grey lines) and the average values (bold line) across the 9 weeks (18 training visits) in N = 3. AMBLE training parameters allow therapists or trainers to personalize ankle dorsiflexion during the gait cycle. These training parameters include (**A**) target angle for peak dorsiflexion (higher being more challenging), (**B**) level of assistance torque that the robot provides (higher challenge with less available assist), (**C**) percent of swing time for rise (toe off to peak swing ankle), and (**D**) percent time holding dorsiflexion.

To better understand the maintenance, improvement or emergence of volitional motor control across multiple joints at 17 months, we investigated the contribution of the individualized robotic training settings on gait biomechanics outcomes. Specifically, we examined the temporal profiles of the AMBLE device inputs (the commanded target dorsiflexion angle, level of assistance (controller stiffness) and dorsiflexion rise and hold times) and their relationships to key device-measured outcomes of ankle–foot control namely, heel-first strikes, peak swing dorsiflexion and dorsiflexion angular velocity. Our analysis revealed that over 18 training visits, on average, the commanded robot setting input changes (post-training versus baseline) in target angle and rise time were increased by 32% and 68%, respectively, while the level of assistance and hold time were decreased by 29% and 56%, respectively (Figure 4). These changes aligned with contemporary principles of motor learning training where a progressive increase in “challenge” (e.g., target angle or angular velocity) is concurrently paired with a progressive decrease in “reliance” (done through a reduction in device assistance) to promote autonomous control of the limb.

**Figure 5:**
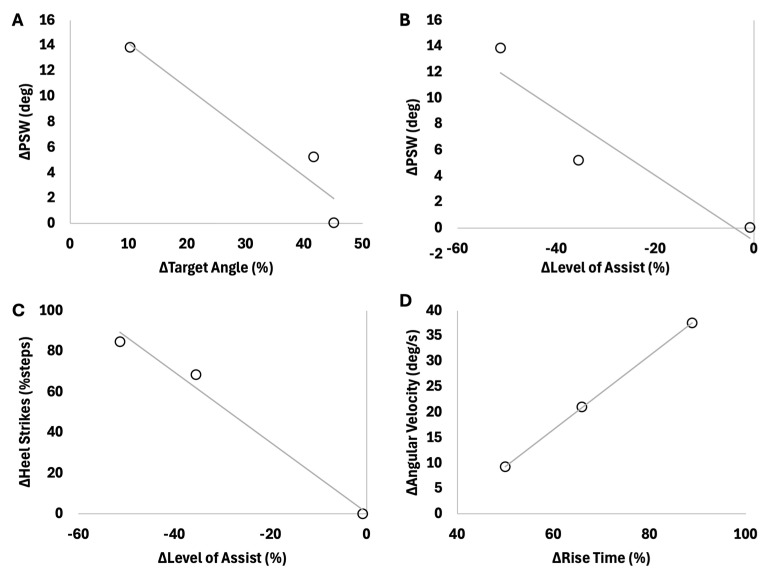
Regression plots showing relationships between the percent change in robot-commanded settings for (**A**) peak dorsiflexion target angle, (**B**,**C**) level of assist and (**D**) rise time versus change in unassisted overground gait biomechanical outcomes for (**A**,**B**) paretic peak swing angle, (**C**) percent steps with heel first landings and (**D**) peak swing angular velocity from baseline to 17 months after training ends in this small sample of individuals. The lines are the regression lines and circles represent the values for the three individuals.

This very small sample size must be interpreted with caution in terms of generalizability. In these three subjects, we found strong correlations between endpoint changes in training inputs and gait biomechanics outcomes (Figure 5). Change in peak swing dorsiflexion exhibited strong negative correlations with changes in target angle (*r*^2^ = −0.96) and level of assistance (*r*^2^ = −0.94) while endpoint change in heel-first strikes was negatively correlated with the level of assistance (*r*^2^ = −0.99). Change in dorsiflexion angular velocity had a strong positive correlation with change in rise time (*r*^2^ = +1.0). These findings were consistent with the intended rehabilitative training objective of weaning device assistance to promote autonomous ankle–foot control, i.e., higher reductions in commanded assistance level resulted in greater gains in volitional unassisted over ground peak swing dorsiflexion and heel-first strikes.

## 4. Discussion

The main finding is that all three individuals with chronic hemiparetic gait and foot drop who completed AMBLE training demonstrated continued improvements in gait biomechanics measures 17 months after training was completed. This temporal profile with initial improvements in ankle–foot function across training is followed later by emerging improvements in ankle, knee and hip kinematics. We hypothesize that this may suggest ongoing locomotor plasticity. The observed collective gait biomechanical improvements are consistent with a safer and more efficient gait pattern, emerging more than a year after robotics training ended. Only gait velocity regresses back to baseline value 17 months after training ends. These findings in persons with severe chronic hemiparetic stroke suggest that dorsiflexion specific adaptive control ankle robotic training improves ankle–foot behavior. This might serve as a stimulus for continued biomechanical improvements in autonomous joint control across the ankle, knee and hip, defining elements of reduced paretic leg pathological joint synergies [27,28,29,30].

Few studies have systematically investigated the durability of treatment effects or for improved biomechanical outcomes following completion of robot assisted gait training. Most studies focus on the short-term changes across training [31,32]. We find that the majority of improvements in gait biomechanics with AMBLE training occur long after completion of training. This differs from prior studies that report that all or most of the gains occur during the robotics-assisted gait training intervention, with selected functional mobility outcomes retained long-term [33,34,35]. A sub-acute stroke robotic-assisted gait training study using an electromechanical suspensory harness end-effector found retained gains in Barthel and Rivermead Mobility Indices, but no further significant improvements two years later [33]. No biomechanical data were reported in this early seminal study. One sub-acute stroke case-controlled study comparing conventional rehabilitation with versus without end-effector robotics-assisted gait training reported improved gait velocity, cadence, paretic single stance time and selected gait biomechanics indexing greater knee and hip efficiency at durability testing [34]. However, the latency between completion of robotic training and durability testing was only 5 weeks, during which time all the participants received ongoing intensive inpatient rehabilitation. This ongoing therapy would be anticipated to augment gait recovery in synergy with the accelerated natural recovery known to occur across the sub-acute stroke period [36]. One non-controlled study enrolling persons with sub-acute and chronic hemiparetic stroke reported end-effector assist-as-needed ankle robotics training over treadmill plus functional electrical stimulation (FES) produced durable improvements in gait velocity, paretic foot clearance and hip joint excursion, but not gait biomechanics, with most gains declining toward baseline by 6 months post-training [35]. We have previously reported in a randomized study of persons with chronic HP stroke and foot drop that treadmill-based adaptive control ankle robotics improves unassisted overground walking velocity, paretic ankle dorsiflexion velocity, peak swing angles and paretic limb propulsion, with all benefits being retained, including continued increases in gait velocity and paretic leg push off 6 weeks after robotics training ended [11]. Thus, our current findings extend prior research that some improvements in gait biomechanics may be durable and even continue to improve in persons with chronic hemiparetic stroke after robotics training has ended.

### 4.1. The Role of Robotic Training Stimuli

Epidemiological studies on the natural history of gait recovery, including clinical rehabilitation and robotics studies, suggest that the sub-acute stroke phase is optimal for neuroplasticity, while the chronic phase yields relatively minimal improvements [5,37]. Our subjects began their robotic therapy a mean of nearly a decade after their index disabling stroke. Therefore, they would not otherwise be expected to further improve after robotics training ended, without any additional gait training stimulus. Hence, we posit that the initial 9 weeks of adaptive control ankle robotics training stimulus is the key determinant in triggering ongoing locomotor plasticity. The observed reductions in training parameter settings for the commanded level of dorsiflexion robotics assist and increases in target peak swing angle represent increased challenge across the 9-week training intervention. The 17-month improvements in ankle peak swing angle and dorsiflexion angular velocity equate to durable improvement in greater proportion of heel first foot landings. Prospective studies in larger numbers are needed to determine whether this post-hoc relationship between ankle robotics prescription progression and long-term improvement in gait biomechanics safety patterning holds up.

We acknowledge that the change in biomechanics could have resulted from many other factors. Daily fluctuations related to fatigue, poor sleep and intercurrent illness could have a negative impact on function and gait biomechanics. The possibility exists that some of the changes in outcomes might be attributable to learning with repeated testing or to measurement variability. To assess the stability of the outcomes’ measures and the plateau of neurologic recovery, each person served as their own pseudo control and had dual baseline testing at least one week apart. There were no statistically significant differences between the two baseline values [15]. Furthermore, many of the training and long-term gains met the meaningful clinically important differences and minimal detectable changes reported in the literature.

None of these three subjects had any interval physical therapy or research interventions after the 9-week robot-assisted gait training to explain the continued late biomechanics gains at 17 months. We were reliant on subject reports of any interval rehabilitation or stroke research study participation. We did not query about any other changes in community participation, changes in lifestyle or new hobbies or activities during the 17-month retention period. The design of the original robot-assisted training study was to improve ankle motor control in the context of a walking task. One possibility for continued improvements is that safer gait patterning and greater walking confidence after training, as observed in our longitudinal outcomes. This may favor increased free-living physical activity. Greater free-living activity auto-reinforces ongoing physical activity dependent plasticity. While gait velocity is currently considered perhaps the most important rehabilitation outcome for persons with stroke [36,37,38], our results suggest that the safety of gait biomechanical patterning is also an important outcome that can be shaped to yield continued improvements up to 17 months after cessation of robotic training, potentially to influence longitudinal outcomes such as free-living mobility and falls.

### 4.2. Potential Mechanisms

This study cannot determine the mechanisms underlying the improved biomechanics. However, the temporal profile of improvements suggests that adaptive-control ankle robotics training produces changes in ankle kinematics across training that lead to long-term distal-to-proximal improvements. At the durability timepoints, gains in peak ankle, knee and hip flexion suggest improved multi-segmental paretic leg sensorimotor control. Hemiparetic stroke has long been recognized to produce pathological synergies consisting of coupled muscle activations across the ankle, knee and hip joints that limit isolated joint control [27]. Meta-analyses of pathologic muscle synergies after stroke and machine learning models on rehabilitative strategies have focused on addressing specific phases of the gait cycle, including foot landing, clearance and push-off in order to facilitate sensorimotor recovery of isolated joint control [28,29]. Our studies show that adaptive control ankle robotics training durably improves heel first strikes, maximum toe clearance, dorsiflexion angular velocity and paretic limb push-off; affecting all of the gait cycle phases that are robustly predicted in ensemble machine learning models to reduce pathological synergies across the paretic limb after hemiparetic stroke [11,15]. Notably, recent motor learning models suggest that the strongest neuromuscular factor influencing pathological synergies after stroke is poorly controlled dorsiflexion throughout the gait cycle [28]. AMBLE is, to our knowledge, the first adaptive control ankle robot enabling real-time precision control across all sub-phases of the dorsiflexion portion of the gait cycle. This shaping of dorsiflexion may contribute, in part, to the unexpected finding of emerging multi-segmental biomechanical improvements to reduce paretic leg pathological joint synergies. Further studies are needed to better understand the mechanisms and temporal profile and the underlying neurophysiological mechanisms of robotics mediated locomotor plasticity after stroke.

### 4.3. Kinematic Patterning Versus Gait Velocity

An unexpected finding is an apparent discrepancy between improvements in biomechanical gait patterning and the absence of sustained improvement in gait velocity. The robot-assisted gait training intervention was conducted at a self-selected walking speed, at a low aerobic intensity. There was no progression of gait speed or aerobic intensity during the 9 weeks of training. AMBLE robotic-assisted training focused on ankle motor control and targeted movement quality of dorsiflexion sub-events. We posit that the observed long-term biomechanical adaptations may be attributed to the task-specific nature of the training stimulus. Its adaptive control algorithms target ground clearance during early swing, bracing control during mid-to-late swing and eccentric control in preparation for ecological landing during late swing. Further, our training protocol focuses on gait biomechanics using precision dorsiflexion assist-as-needed training inputs, not velocity-based training. This control version of AMBLE does not target plantar flexion. Since plantar flexion is the main driver of gait propulsion, we would not predict gains in gait velocity. Thus, the nature of the delayed adaptations may be task-specific to the dorsiflexion training prescription. Alternatively, people living with hemiparetic gait and footdrop may autonomously trend toward improved biomechanical gait safety over faster walking speed. Targeting faster gait velocity has been associated with greater fall risk, which could increase fear of falling and behaviorally reinforce slower, safer walking patterns [36,37].

### 4.4. Clinical Implications

Ankle-robotic-assisted gait rehabilitation can improve paretic ankle–foot behavior to promote longitudinal locomotor recovery long after the precedent training ends. A novel chronic stroke management model in which a relatively short course of precision ankle robotics training could prime ongoing neuroplasticity to help shape gait recovery. Improved and safer gait patterns may promote greater free-living ambulatory activity. Such a model with precedent robotic gait training superimposed on current recommendations for physical activity and/or prescribed functional mobility exercises after stroke could accelerate the biomechanical, physiological and whole-health benefits for persons aging with the chronic disability of stroke [39,40,41,42]. More broadly, these findings should inform multi-disciplinary rehabilitation providers to look not just at the short-term pre-post changes of time-delimited therapeutics, such as robotics, but also the longitudinal after-effects and ongoing physical activity behaviors that influence long-term rehabilitation outcomes in this population.

### 4.5. Limitations

Since only 3 of the original 24 participants were available serendipitously for the 17-month follow-up, these findings cannot be generalized to the broader hemiparetic stroke population. We do not systematically track free-living ambulatory activity, community-based therapy or intercurrent health events, so we cannot attribute ongoing improvements solely to prior AMBLE training. Other behavioral factors, such as higher levels of free-living ambulatory activity after robotics training was completed, may have influenced the longitudinal outcomes. Another limitation is the fact that all three of these participants have more severe hemiparesis and foot drop many years after their index disabling stroke. This raises the possibility that the delayed improvements after robotics training ends may be unique or more likely for people with more severe or chronic neurological deficits. It is likely that individuals with greater deficits simply require longer adaptation times to manifest improvement up the kinematic chain. Accordingly, the present results may be viewed as only hypothesis-generating. Regardless, the findings are significant in drawing attention to the potential for emerging biomechanical improvements out to a year or more after robotics training ends, and awareness that novel mechanisms of neuroplasticity involving reduction in pathological joint synergies warrants further study.

## 5. Conclusions

In summary, this brief report finds that the majority of improvements in gait biomechanics attributable to dorsiflexion-specific adaptive control ankle robotics do not occur across training but occurred during the ensuing 17-month durability testing period in persons with chronic hemiparesis and foot drop after stroke. The temporal profile of initial improvements in ankle–foot patterning is followed by emerging biomechanical improvements in ankle, knee and hip kinematics out to as much as 17 months after training. These ongoing improvements are consistent with reduced pathological joint synergies. Larger randomized studies are needed to determine the optimizing dosage(s) and underlying neuroplastic mechanisms for adaptive control ankle robotics to improve long-term functional mobility and prevent falls for persons that have suffered a hemiparetic stroke.

## Figures and Tables

**Table 1:** Baseline demographic and clinical characteristics of N = 3 subjects.

Characteristic/Study ID	CPTR16	CPTR20	CPTR25
Age (years)	52	65	69
Time post-stroke (months)	95	25	176
Gender (M/F)	M	F	F
Height (cm)	165	150	170
Weight (kg)	74	63.5	75
Paretic side (L/R)	L	L	L
AFO use (Y/N)	N	Y	Y
Assistive device use (Y/N)	SPC	QC	SPC
MMT-DF (0–5)	2+	2−	1+
Active ROM-DF (°)	−15	−5	−15
Passive ROM-DF (°)	−15	0	−5
Gait velocity (m/s)	0.56	0.49	0.57
DGI score (0–24)	12	14	15
6 MWT distance (meters)	184.6	152.4	289.6

M/F: male/female; cm: centimeter; kg: kilogram; L/R: left/right; AFO: ankle foot orthotic; Y/N: yes/no; MMT-DF: manual muscle test score in dorsiflexion; ROM: range of motion; °: degree; m/s: meters per seconds; DGI: dynamic gait index; 6 WMT: six-minute walk test.

## Data Availability

Information on this clinical trial (Clinical Trial Identifier: NCT04594837). Information can be found at: https://clinicaltrials.gov/study/NCT04594837 (accessed on 21 April 2026). The datasets associated with the study are not publicly available due to protection of participant privacy policy. A minimal dataset can be made available in a de-identified fashion from the corresponding authors, upon a reasonable request, without investigator support, after approval of the proposal by the University of Maryland Baltimore and NextStep Robotics team, and with a signed data access agreement.

## Supplementary Materials

The following supporting information can be downloaded at: https://www.mdpi.com/article/10.3390/medicina62071250/s1, Table S1: Baseline Characteristics of the Original Cohort.

## Abbreviations

3-D	Three dimensional
6MWT	Six-minute walk test
17M	17-month retention period
°	Degree
°/s	Degree per seconds
∆	Delta
%	Percentage
% steps	Percent steps
A/D	Assistive device
AFO	Ankle foot orthosis
cm	Centimeter
deg	Degree
deg/s	Degree per seconds
DF	Dorsiflexion
DOF	Degree of freedom
DGI	Dynamic Gait Index
e.g.	For example
F	Female
HF	Heel-first
kg,	Kilogram
L	Left
M	Male
m/s	Meters per second
MMT-DF	Manual muscle test in dorsiflexion;
N	No and number
NIH	National Institute of Health
NINDS	National Institute of Neurological Disorders and Stroke
Nm	Newton meters
P	Paretic
PSW	Peak swing angle
QC	Quad cane
R	Right
r^2^	Coefficients of determination
ROM-DF	Range of motion in dorsiflexion
SD	Standard deviation
SPC	Single point cane
TPS	Time post-stroke
Vs	Versus
Y	Yes
