# Supplementary material for: Ankle Robotics Induces Ongoing Locomotor Plasticity with Delayed, Sustained Multi-Segmental Gait Improvements 17 Months After Training in Chronic Stroke
Source: Medicina (Kaunas). 2026 Jun 29;62(7):1250. doi: 10.3390/medicina62071250 (PMC13414436; doi:10.3390/medicina62071250)
Supplement: Supplementary file 1 [file medicina-62-01250-s001.zip › medicina-4301712-supplementary.pdf]

**Supplementary Table S1.** Demonstrates the Baseline Characteristics of the Three Participants at the 17-Month Durability Testing from the Parent Study (N=24 Subjects Completed the Parent Study) Table 3 from JNER 2025 [15].

| ID | Age (yr.) | TPS (mos.) | Gender | Paretic Side | AFO | AD | MMT | AROM (deg) | Speed (m/s) | Ambulation Category* |
|---|---|---|---|---|---|---|---|---|---|---|
| 1 | 76 | 266 | F | R | Y | Y | 1+ | -19 | 0.370 | H |
| 2 | 37 | 235 | F | R | Y | N | 4- | -17 | 0.970 | C |
| 4 | 70 | 19 | M | L | N | 4PC | 4- | -5 | 0.460 | LC |
| 5 | 49 | 36 | M | R | N | N | 4- | -13 | 0.929 | C |
| 6 | 68 | 134 | F | R | Y | N | 4- | -8 | 0.550 | LC |
| 7 | 44 | 14 | F | L | N | 1PC | 4- | -3 | 0.620 | LC |
| 8 | 34 | 19 | F | L | Y | N | 4- | -14 | 0.699 | LC |
| 9 | 58 | 86 | M | L | N | 1PC | 4- | -5 | 0.785 | LC |
| 10 | 30 | 360 | F | L | Y | N | 1 | 0 | 1.000 | C |
| 11 | 60 | 233 | M | L | Y | 1PC | 4- | -19 | 0.844 | C |
| 12 | 55 | 138 | M | R | Y | N | 4- | 5 | 0.796 | LC |
| 14 | 47 | 56 | F | L | Y | 1PC | 1 | 0 | 0.483 | LC |
| 16 | 52 | 95 | M | L | N | 1PC | 2+ | -15 | 0.603 | LC |
| 18 | 39 | 66 | M | R | Y | 1PC | 0 | -25 | 0.738 | LC |
| 20 | 65 | 25 | F | L | Y | 4PC | 2- | -5 | 0.510 | LC |
| 21 | 64 | 308 | M | L | N | 1PC | 4+ | 0 | 0.945 | LC |
| 22 | 68 | 17 | M | L | Y | 1PC | 2- | -10 | 0.740 | LC |
| 23 | 58 | 24 | M | R | N | 4PC | NA | NA | 0.689 | LC |
| 24 | 70 | 56 | M | R | Y | 4PC | 1 | 0 | 0.211 | H |
| 25 | 69 | 176 | F | L | Y | 1PC | 1+ | -15 | 0.771 | LC |
| 26 | 67 | 210 | F | L | N | 4PC | 1+ | -20 | 0.291 | H |
| 27 | 57 | 100 | F | L | N | 1PC | 3- | 0 | 0.826 | C |
| 29 | 66 | 31 | M | R | N | 4PC | 1 | -5 | 0.421 | LC |
| 30 | 53 | 37 | F | L | Y | 4PC | 1 | -5 | 0.144 | H |

TPS: time post-stroke, AFO: ankle foot orthotic, AD: assistive device, MMT: manual muscle test in dorsiflexion, DGI: Dynamic Gait Index, AROM: active range of motion in dorsiflexion, 4PC: quad cane, 1PC: single point cane. N: no device. *Ambulation category based on walking speed: (H: home gait speed ≤ 0.4 m/s; LC: limited community > 0.4 m/s to ≤ 0.8 m/s; C: community ambulator > 0.8 m/s) [19].
